# Perception of clinical educational environment by student of physiotherapy based on the Postgraduate Hospital Educational Environment Measurement Questionnaire in Chile

**DOI:** 10.3352/jeehp.2019.16.16

**Published:** 2019-06-14

**Authors:** Karen Córdova-León, Lincoyán Fernández-Huerta, Marcela Rojas-Vargas

**Affiliations:** 1 Kinesiology School, Faculty of Health Sciences, Universidad de las Américas, Concepción, Chile; 2 Doctorate School, Faculty of Physical Therapy, Universitat de València, Spain; Hallym University, Korea

**Keywords:** Educational environment, Student perception, Professional education, Academic medical centers, Surveys and questionnaires, Chile

## Abstract

**Purpose:**

It aimed at describing the perception of the clinical educational environment by physiotherapy students based on the Postgraduate Hospital Educational Environment Measurement Questionnaire in Chile.

**Methods:**

The clinical education environment was evaluated according to the Postgraduate Hospital Educational Environment Measure (PHEEM) by 192 students originally enrolled in the fifth year of the physiotherapy career at 3 different headquarters of the academic institution: Santiago, Viña del Mar, and Concepcion Campus (Metropolitan, Valparaiso, and Bio Bio region, respectively), from March to October 2018. The Cronbach’s α was applied to measure the reliability of the instrument and the Student-t and analysis of variance tests were used to compare the differences of PHEEM scores by headquarters, environmental areas, and experience of internship.

**Results:**

A total overall average score of 125.88 was obtained, which meant an excellent educational environment. The overall score was 127.6±22.7 for headquarters 1, 125.6±21.6 for headquarters 2, and 122.5±26.9 for headquarters 3. According to the type of establishment, the scores were of 127.1±22.1 for private and 123.5±26.3 for public institutes. According to the type of area, the score was cataloged as an excellent educational environment in all cases, except in the respiratory care area (lowest score, 117.5±29.1). Finally, the score was 126.9±20.5 for the first internship, 121.7±29.3 for the second, and 129.4±19.6 for the third.

**Conclusion:**

There is relative homogeneity of the clinical educational environment for different headquarters, types of establishment, or type of area; but there are significant differences in the number of the internship. The promotion of a good clinical educational environment can have an important impact on the development and performance of the future professional, being the detection of negative aspects an opportunity to improve the hidden curriculum.

## Introduction

Is highly accepted that the educational environment has a strong influence on the learning process, social life, and future work. The performing of the educational environment includes a great variety of factors such as student welfare, the quality of teaching, the curriculum, the perception of academic achievement, the number of learning opportunities, facilities available, and others which together attribute to each educational institution a differentiating aspect [1].

Students’ perceptions of the educational environment play a decisive role in the planning and implementation of a curriculum. It also helps stakeholders and the schools themselves to reflect, reform, and remedy to make the curriculum friendly for students without compromising or detracting from the standards and quality of the teaching-learning process [2]. Therefore, feedback and systematic evaluation are vital for the successful management of the curriculum. The evaluation of the environment in contexts of practical courses, which usually have many more variables to consider, may help to solve educational problems and improve the effectiveness of the process [3]. According to the study of Fuenzalida et al. [4], the evaluation of the clinical educational environment contributes to identify the main strengths and aspects that may be improved, which also serve as the basis for developing a future action plan that could directly benefit students, academics, and other stakeholders. This understanding about that the educational perception is a reflective observation of the student about his educational environment and that can help to optimize the environment is vital and an important contribution for the measurement to be considered part of the internal self-evaluation process for each career and also a contribution to the accreditation process [5].

There are many instruments have been translated from the English and subsequently validated to other different languages, being reflect of the interest and relevance of the continuous evaluation of the educational environment for the academic community [6], a trend based on the need to obtain objective measures supporting education based on results [6]. In the clinical field, questionnaires such as the Postgraduate Hospital Educational Environment Measure (PHEEM) have been created, which one was originally manufactured for the medical career and with language adaptation for their own specialties [7]. There is no specific instrument of evaluating educational environment created for rehabilitation careers, for example, physiotherapy. It is recognized that a wide knowledge of the educational environment facilitates the possibilities of approach, allowing a better evaluation of one of the many aspects present in the academic formation. For this reason, the objective of this study was to describe the physiotherapy students’ perception of educational clinical environment by adopting the PHEEM Questionnaire.

## Methods

### Ethics statement

The Institutional Ethical Committee at Universidad de las Américas approved the study protocol (UA126). The informed consent was contained in the questionnaire form.

### Study design

It is the descriptive and observational study.

### Materials and/or subjects

The PHEEM Questionnaire has shown to have good psychometric results of validity and reliability [8]. The original instrument consists of 40 items, contains 36 positive affirmations, and 4 negative ones. It has a scale from ‘totally in disagreement’ to ‘totally agree,’ with a score of 0 to 4 on a Likert scale, grouped into 3 subscales for perceptions of role autonomy, teaching, and social support. The translation and validation used as the basis for this study, retains the same characteristics of the original, but with modifications in technical concepts typical of the physiotherapy career. This adaptation was made with the supervision, suggestions, and authorization of the original authors (Tables 1, 2). The higher the score, the better the perception of the clinical educational environment.

### Technical information

A total of 419 questionnaires were obtained from 192 students originally enrolled in the fifth year of the physiotherapy career at 3 different headquarters of the academic institution: Santiago, Viña del Mar, and Concepcion Campus (Metropolitan region, Valparaiso region, and Bio Bio region, respectively). The number of interns varied in each internship due to situations typical of the progression in the curriculum of a student. The questionnaire was applied in 3 internships of the professional practical subject, in the last week of each one (from March to October 2018). These internships do not have a specific thematic area in order, they are assigned at random. The only requirement is to incorporate an internship in each discipline area of their clinical training. The questionnaire was administered through web-based form, which also where students allowed the use of the data voluntarily, anonymously, and confidentially.

### Statistics

The original scale of interpretation of results was used [8]. The PHEEM Questionnaire can also be used to pinpoint more specific strengths and weaknesses within the educational climate and to do this one needs to observe at the responses to individual items, but it was not the aim of the study. The Cronbach’s α was calculated to evaluate the internal consistency of the PHEEM Questionnaire results, for both the total mean. Descriptive measures were obtained for each domain of the scale and the results were stratified by internship number, headquarter, and type of area. The Kolmogorov-Smirnov test was used to evaluate the normality of the data. Analysis of variance (one-way analysis of variance test) and mean comparison (Student t-test) was used to determine the significance of differences among internship number, headquarter, and type of area, with a level of significance set at P<0.05. Descriptive analysis was performed to calculate the mean score for each item; these item scores were then used to identify problem areas as a whole and by the number of internships. Descriptive and inferential analysis was performed using IBM SPSS ver. 21.0 (IBM Corp., Armonk, NY, USA).

## Results

We received 419 completed questionnaires. The PHEEM Questionnaire adapted for the physiotherapy career obtained a Cronbach’s α of 0.90, proving to have excellent reliability. The raw data are available in Supplement 1.

The overall perception of the clinical educational environment had a score of 125.9±23.6 (excellent educational environment), the perception of the role of autonomy was 35.7±5.9 (a more positive perception of the role of each), the perception of teaching quality was 51.7±11.8 (model teachers), and social support was 31.1±6.3 (more pro than contras). Descriptive summaries in Table 3.

When comparing the results according to the number of the internship, the number of responses was homogeneous for the descriptive measures. The overall score decreased from the first to second internship and then increased from the second to the third internship, the latter being the highest score of the three and the second the lowest. This occurs in all domains of the questionnaire (Table 3). The differences were statistically significant in the overall score and all its domains (P<0.05) (Table 4).

Regarding headquarters, there are differences in the descriptive measures obtained, but they were not statistically significant in any of the domains (Table 4). The overall score was 127.6±22.7 for headquarters 1, 125.6±21.6 for headquarters 2, and 122.5±26.9 for headquarters 3, all of which were classified as having an ‘excellent educational environment’ (Table 3).

When comparing the types of establishment, the overall scores were 127.1±22.1 for private and 123.5±26.3 for public, the environments of both types of establishments were cataloged with an ‘excellent educational environment’ (Table 3). The differences were not statistically significant in any of the domains (Table 4).

When comparing the environment in the different areas of sanitary rehabilitation, the overall score was cataloged as an ‘excellent educational environment’ in all cases, except in the ‘Sala ERA’ (respiratory care room; lowest score, 117.5 ±29.1; educational environment more positive than negative with space for improvement). The highest score was found in ‘MAS program’ (care of self-heating elderly people) with 135±16.9 (Table 3). The differences were not statistically significant in any of the domains (Table 4); however, the fact of the homogeneity of distribution of responses must be considered (Table 4).

## Discussion

This study was conducted to describing the perception of the clinical educational environment by physiotherapy students based on the Postgraduate Hospital Educational Environment Measurement Questionnaire in Chile.

A relative homogeneity of the results was found with similar scores for the different sites, types of establishment, or rehabilitation areas. But, there are significant differences in the number of internships, showing an increase in the score as they advance, therefore, improve the perception of the clinical educational environment. This, could reflect the perception of preparation and accommodation on the part of the students to face the changes of scenarios [9], something that is also consistent with the progression of the stages declared in the course’s own program of the race where this study was implemented (stage 1, introduction to the service; stage 2, introduction to the professional role; stage 3, empowerment of the professional role; and stage 4, autonomy of the professional role), which speak of a progression in the time the practice is developed and where the student is expected to acquire challenges and graduated activities, being evaluated as expected in each of the cycles. The variation of the score on this subscale could also be supported by the empirical evidence that indicates that students initiate a training program with a higher expectation of their educational learning environment are likely to have more general perceptions of an environment throughout the course of the course of the program [10].

The literature has reported variations depending on the type of clinical internship attended by the student and the type of training institution in which he performs his training [11]. Galli et al. [12] mention that the perception of the interns is less favorable in the public sector. In Chile, there is a public regulatory framework that could present deficiencies. It is the National Teaching Assistance Commission (CONDAS) that defines the clinical field as a healthcare facility with adequate conditions of structure, personnel, and equipment and it commits to promote the advancement of the disciplinary and generic competences defined by health careers for its graduates [13]. In more qualitative analysis, Galli et al. [12], mention aspects that make the difference are: having a continuous clinical supervision, having the conditions in the health facilities of work and rest, feeling the sensation of physical security within the establishment, and having the flow adequate daily attendance with respect to the time of permanence in practice [14]. Due to the influence that the indicated legal and administrative changes can have on the interrelation of teaching care in the public sector, it is fundamental that the state organisms and training centers are able to assume shared responsibilities for a better understanding of the problems that arise in the development of the practices.

The general result of the questionnaire was an ‘excellent educational environment’ in each internship (first, second, and third) carried out by the intern. However, when observing the type of area, the ‘Sala ERA’ (respiratory care room) has a lower average score than the rest and outside the maximum range of interpretation of the scale. When observing the scores of the domains, the social support is under the maximum range, which shows the possibility of improving some points with respect to the conditions of the centers where these presses are developed.

The perception domains of the clinical educational environment for the quality of education and global scoring were within maximum ranges, but not for the role of autonomy and social support, so there is space for improvement for both aspects considering that the first could depend more on intrinsic characteristics of the intern and the second one, could depend on external factors. The global score could be due to recent curricular modification by the career that deepens the educational model of the institution centered on the student, seeking to prepare it to achieve an autonomous role of the profession gradually by facilitating the sense of identity, generation of habits, and norms that specify the institutional culture based on the values of professional ethics, civic responsibility, and community commitment. In any case, Quiroga-Maraboli et al. [15] recommends complementing the findings of the different questionnaires that evaluates the perceptions of the educational environment with a qualitative study to explore the current context and to propose potential solutions to the problematic subscales or each element.

For the autonomy role, the results seem to be consistent with other investigations where the internal or resident students have experienced personal growth as time goes by in their practice activities [12]. According to Clouder and Adefila [14], the level of confidence of the student is associated with the ability to learn to the extent that they are empowered to assume increasing levels of responsibility, and that this in turn depends to a large extent on active participation in that practice [16]. Clinical supervision is an important factor to avoid the fluctuating self-confidence that students refer to move towards autonomous practice [17].

Though this study has some limitations, for example having sample from one specific region from Latin America and only from one educational institution thus difficult to generalize for the others that could be addressed to some extent by comparison of the results with other studies. However, it is suggested to conduct the similar study on a large scale and covers as many as possible regions and schools from physiotherapy.

We do not declare demographic variables such as sex, age, and cultural background, which could be considered an inconvenience. However, the purpose of the study was to describe the perception of the clinical educational environment by physiotherapy students based on the Postgraduate Hospital Educational Environment Measurement Questionnaire as an approach to the diagnosis and use it as one of the improvement materials of the same and other areas of the career curriculum.

The students are a pertinent source of information. However, the perception of the educational environment among different groups of students is idiosyncratic and may differ widely from 1 year to the next, so a cohort follow-up would be necessary. Likewise, it is necessary to be clear about which students are only one part of the educational scenario, so that the perceptions of teachers and other stakeholders are equally important. Future studies may be focused in the multipart that make up the clinical education environment.

To conclude, we believe that the measurement of the clinical educational environment should be recognized as an important edge in the self-assessment process of careers and institutions to assess the impact of changes in clinical practice and curricular innovation processes. Considering other studies that have also used this questionnaire, we emphasize that it should be used in its original format with the 40 basic questions to allow comparisons between programs and allow evaluations of the 3 domains during the different phases of the professional practice, considering the adaptations of technical language for each health discipline.

## Figures and Tables

**Table 1.** PHEEM Questionnaire modified and applied to students of physiotherapy (in Spanish)

No.	PHEEM Questionnaire
1.	Conozco el programa de asignatura que provee información sobre las actividades de Práctica Profesional.
2.	Mi(s) tutor(es) establece(n) expectativas claras sobre lo que esperan de mi desempeño.
3.	Tengo el tiempo suficiente para preparar mis actividades académicas.
4.	Tuve un plan de inducción informativo.
5.	Tengo el nivel adecuado de responsabilidad en esta rotación.
6.	Tengo supervisión clínica adecuada en todo momento de parte de mi(s) tutor(es).
7.	Hay discriminación cultural y religiosa en esta rotación.
8.	Tengo que realizar tareas que no corresponden a un interno de kinesiología.
9.	Hay un manual normativo de conductas profesionales para los internos.
10.	Mi(s) tutor(es) tiene(n) buenas habilidades de comunicación con los internos.
11.	Soy interrumpido de manera inapropiada durante mi trabajo de interno.
12.	Puedo participar activamente en otras actividades educativas.
13.	Hay discriminación por sexo en esta rotación.
14.	Tengo guías claras acerca de mis actividades en esta rotación.
15.	Mi(s) tutor(es) es(son) entusiasta(s).
16.	Tengo una buena colaboración con mis compañeros.
17.	Mi horario se ajusta al horario previamente informado.
18.	Tengo la oportunidad de seguir la evolución de los usuarios y/o comunidades.
19.	Tengo de parte de mi(s) tutor(es) una guía adecuada en mi trabajo como interno.
20.	Este establecimiento tiene una buena calidad de espacios físicos para desarrollar actividades docentes.
21.	Hay acceso a un programa educativo pertinente a mis necesidades.
22.	Recibo retroalimentación periódica de mi(s) tutor(es).
23.	Mi(s) tutor(es) está(n) bien organizado(s) en sus actividades académicas.
24.	Me siento físicamente seguro en el centro de Práctica Profesional.
25.	Hay una cultura de tolerancia frente a las equivocaciones que pueda cometer en Práctica Profesional.
26.	Existen instalaciones adecuadas para la alimentación.
27.	Tengo la suficiente oportunidad de aprender para mis necesidades del ámbito profesional.
28.	Mi(s) tutor(es) tiene(n) habilidades para la enseñanza y destreza clínica kinésica.
29.	Aquí me siento parte de un equipo de trabajo.
30.	Tengo la oportunidad de adquirir los procedimientos adecuados para la práctica kinesiológica.
31.	Mi(s) tutor(es) está(n) disponible(s) para resolver mis dudas.
32.	Mi carga de trabajo en esta rotación es adecuada.
33.	Los docentes utilizan las oportunidades de aprendizaje en forma efectiva.
34.	La formación en esta Práctica Profesional me hace sentir listo para la profesión de kinesiólogo.
35.	Mi(s) tutor(es) tiene(n) buenas destrezas como mentor (persona que, con mayor experiencia o conocimiento, ayuda a una persona de menos experiencia o conocimiento).
36.	Tengo tiempo de disfrute en las actividades de esta rotación.
37.	Mi(s) tutor(es) me animan a ser un estudiante independiente.
38.	Hay oportunidades de reforzamiento en caso de obtener resultados insatisfactorios.
39.	Mi(s) tutor(es) me retroalimenta(n) sobre fortalezas y debilidades.
40.	Mi(s) tutor(es) promueven una atmósfera de mutuo respeto.

PHEEM, Postgraduate Hospital Educational Environment Measure.

**Table 2.** PHEEM Questionnaire modified and applied to students of physiotherapy (adaptation translate to English)

No.	PHEEM Questionnaire
1.	I know the subject program that provides information about the activities of Professional Practice.
2.	My tutor(s) sets clear expectations about what they expect from my performance.
3.	I have enough time to prepare my academic activities
4.	I had an information induction plan.
5.	I have the appropriate level of responsibility in this rotation.
6.	I have adequate clinical supervision at all times from my tutor(s).
7.	There is cultural and religious discrimination in this internship.
8.	I have to perform tasks that do not correspond to a physiotherapy intern.
9.	There is a normative manual of professional behaviors for internships.
10.	My tutor(s) have good communication skills with intern.
11.	I am interrupted inappropriately during my intern work.
12.	I can actively participate in other educational activities.
13.	There is discrimination by sex in this rotation.
14.	I have clear guidelines about my activities in this internship.
15.	My tutor(s) is(are) enthusiastic(s).
16.	I have a good collaboration with my colleagues.
17.	My schedule is adjusted to the previously informed schedule.
18.	I have the opportunity to follow the evolution of the users and/or communities.
19.	I have an appropriate guidance from my tutor(s) in my work as an intern.
20.	This establishment has a good quality of physical spaces to develop teaching activities.
21.	There is access to an educational program relevant to my needs in this practice.
22.	I receive periodic feedback from my tutor(s).
23.	My tutor(s) is(are) well-organized in their academic activities.
24.	I feel physically secure in the professional practice center.
25.	There is a culture of tolerance against the mistakes that can be made in professional practice.
26.	There are adequate facilities for food.
27.	I have enough opportunity to learn for my professional needs.
28.	My tutor(s) has(have) skills for teaching and clinical physical therapy skills.
29.	Here I feel part of a work team.
30.	I have the opportunity to acquire the appropriate procedures for physiotherapy practice.
31.	My tutor(s) is(are) available to answer my questions.
32.	My workload in this internship is adequate.
33.	Teachers use learning opportunities effectively.
34.	The training in this professional practice makes me feel ready for the profession of physical therapist.
35.	My tutor(s) has(have) good skills as a mentor (person who, with more experience or knowledge, helps a person with less experience or knowledge).
36.	I have time to enjoy the activities of this rotation.
37.	My tutor(s) encourages(encourage) me to be an independent student.
38.	There are opportunities for reinforcement if unsatisfactory results are obtained.
39.	My tutor(s) gives(give) me feedback on strengths and weaknesses.
40.	My tutor(s) promotes(promote) an atmosphere of mutual respect.

PHEEM, Postgraduate Hospital Educational Environment Measure.

**Table 3.** Descriptive measures of the PHEEM Questionnaire domains

Variable	No. of cases	Domain of the PHEEM survey	Min	Max	Mean±SD
Total of responses	419	Perceptions of role autonomy	8	48	35.7±5.9
		Perceptions of teaching	0	60	51.7±11.8
		Perceptions of social support	6	44	31.1±6.3
		Global score	24	160	125.9±23.6
No. of internship					
First	176	Perceptions of role autonomy	8	47	35.6±5.4
		Perceptions of teaching	0	60	52.4±10.1
		Perceptions of social support	12	43	31.5±5.5
		Global score	24	154	126.9±20.5
Second	135	Perceptions of role autonomy	12	48	34.8±7.0
		Perceptions of teaching	1	60	49.4±14.6
		Perceptions of social support	6	44	30.3±7.6
		Global score	24	160	121.7±29.3
Third	108	Perceptions of role autonomy	22	48	36.9±4.8
		Perceptions of teaching	12	60	53.4±9.9
		Perceptions of social support	9	44	31.7±5.7
		Global score	50	160	129.4±19.6
Headquarter					
One	233	Perceptions of role autonomy	8	48	36.1±5.7
		Perceptions of teaching	0	60	52.3±11.6
		Perceptions of social support	12	44	31.7±6.0
		Global score	24	160	127.6±22.7
Two	93	Perceptions of role autonomy	12	48	34.8±5.3
		Perceptions of teaching	1	60	52.4±10.5
		Perceptions of social support	8	44	31.0±6.1
		Global score	24	160	125.6±21.6
Three	103	Perceptions of role autonomy	16	48	35.6±6.7
		Perceptions of teaching	8	60	49.7±13.0
		Perceptions of social support	6	44	30.1±7.1
		Global score	35	160	122.5±26.9
Type of establishment					
Private	279	Perceptions of role autonomy	8	48	35.8±5.4
		Perceptions of teaching	0	60	52.2±11.2
		Perceptions of social support	9	44	31.6±5.9
		Global score	24	160	127.1±22.1
Public	140	Perceptions of role autonomy	12	48	35.4±6.7
		Perceptions of teaching	1	60	50.7±12.9
		Perceptions of social support	6	44	30.3±7.0
		Global score	24	160	123.5±26.3
Type of area					
Multiarea	279	Perceptions of role autonomy	8	48	35.8±5.4
		Perceptions of teaching	0	60	52.2±11.2
		Perceptions of social support	9	44	31.6±5.9
		Global score	24	160	127.1±22.1
CCR	32	Perceptions of role autonomy	12	48	35.3±7.3
		Perceptions of teaching	1	60	50.0±15.0
		Perceptions of social support	8	44	31.0±7.6
		Global score	24	160	123.6±30.5
ERA	51	Perceptions of role autonomy	17	44	34.0±6.6
		Perceptions of teaching	8	60	47.9±14.9
		Perceptions of social support	6	39	28.7±7.8
		Global score	35	144	117.6±29.2
IRA	46	Perceptions of role autonomy	16	48	36.4±6.5
		Perceptions of teaching	18	60	52.7±9.0
		Perceptions of social support	14	44	31.1±5.3
		Global score	52	160	127.4±20.2
Community Based Rehabilitation	1	Perceptions of role autonomy	38	38	38.0±0.0
		Perceptions of teaching	57	57	57.0±0.0
		Perceptions of social support	29	29	29.0±0.0
		Global score	132	132	132.0±0.0
MAS	10	Perceptions of role autonomy	26	48	37.7±6.1
		Perceptions of teaching	47	60	56.9±5.0
		Perceptions of social support	20	44	32.7±6.8
		Global score	106	160	135.0±16.9

PHEEM, Postgraduate Hospital Educational Environment Measure; Min, minimum value found; Max, maximum value found; Mean, arithmetic mean value of the sample; SD, standard deviation of the sample; Sala CCR, community rehabilitation center for neuromusculoskeletal diseases; Sala ERA, chronic respiratory care room for adults; Sala IRA, acute respiratory infections care room in children; RBC, community-based rehabilitation; MAS, health promotion program for the elderly.

**Table 4.** Comparison of the domains of the survey according to the number of the internship, headquarter, the type of establishment (private sector or public sector), and type of area or specialty (ERA, IRA, CCR, RBC, and MAS program)

Domain of the survey	No. of partnership	Headquarters	Type of establishment	Type of area
Perceptions of role autonomy	0.006^*^	0.184	0.465	0.255
Perceptions of teaching	0.001^*^	0.149	0.203	0.104
Perceptions of social support	0.002^*^	0.103	0.055	0.087
Global score	0.001^*^	0.189	0.15	0.107

Sala ERA, chronic respiratory care room for adults; Sala IRA, acute respiratory infections care room in children; Sala CCR, community rehabilitation center for neuromusculoskeletal diseases; RBC, community-based rehabilitation; MAS, health promotion program for the elderly.

Statistical significance *P<0.05. One-way analysis of variance for more than 2 samples and Student t-test for 2 samples comparison.

## Supplementary materials

Supplement 1. Data files are available from https://doi.org/10.7910/DVN/SHWNK1.

Supplement 2. Audio recording of the abstract.
